# Heparin Inhibits Hepatocyte Growth Factor Induced Motility and Invasion of Hepatocellular Carcinoma Cells through Early Growth Response Protein 1

**DOI:** 10.1371/journal.pone.0042717

**Published:** 2012-08-13

**Authors:** Evin Ozen, Aysim Gozukizil, Esra Erdal, Aykut Uren, Donald P. Bottaro, Nese Atabey

**Affiliations:** 1 Department of Medical Biology and Genetics, School of Medicine, Dokuz Eylul University, Izmir, Turkey; 2 Lombardi Comprehensive Cancer Center, Georgetown University Medical Center, Washington, D.C., United States of America; 3 Urologic Oncology Branch, Center for Cancer Research, National Cancer Institute, National, Institutes of Health, Bethesda, Maryland, United States of America; Aix-Marseille University, France

## Abstract

The Hepatocyte Growth Factor (HGF)/c-Met signaling pathway regulates hepatocyte proliferation, and pathway aberrations are implicated in the invasive and metastatic behaviors of hepatocellular carcinoma (HCC). In addition to c-Met, heparin acts as a co-receptor to modulate pathway activity. Recently, anti-metastatic and anti-cancer effects of heparin have been reported. However, the role of heparin in the regulation of HGF signaling remains controversial and the effects of heparin on HGF-induced biological responses during hepatocarcinogenesis is not yet defined. In this study we determined the effects of heparin on HGF-induced activities of HCC cells and the underlying molecular mechanisms. Here, we report for the first time that heparin inhibits HGF-induced adhesion, motility and invasion of HCC cells. In addition, heparin reduced HGF-induced activation of c-Met and MAPK in a dose-dependent manner, as well as decreased transcriptional activation and expression of Early growth response factor 1 (Egr1). HGF-induced MMP-2 and MMP-9 activation, and MT1-MMP expression, also were inhibited by heparin. Stable knockdown of Egr1 caused a significant decrease in HGF-induced invasion, as well as the activation and expression of MMPs. Parallel to these findings, the overexpression of Egr1 increased the invasiveness of HCC cells. Our results suggest that Egr1 activates HGF-induced cell invasion through the regulation of MMPs in HCC cells and heparin inhibits HGF-induced cellular invasion via the downregulation of Egr1. Therefore, heparin treatment might be a therapeutic approach to inhibit invasion and metastasis of HCC, especially for patients with active HGF/c-Met signaling.

## Introduction

Hepatocellular carcinoma (HCC) is the most common form of primary liver cancer and the third leading cause of cancer-related deaths worldwide [1]. The lethality of HCC is partly associated with its resistance to currently available anticancer agents and a lack of biomarkers that can detect the early stages of the disease. Although liver transplantation and surgical resection improves overall survival in patients with small, non-invasive and non-metastatic tumors, there is still no effective treatment for patients with advanced disease [2], [3].

In addition to the clinical and histopathological characteristics of advanced HCC, recent data have shown that HGF and its high affinity receptor tyrosine kinase c-Met are implicated in the development and progression of HCC [4]. Furthermore, it has been demonstrated that HCC patients with active HGF/c-Met signaling have a significantly worse prognosis and higher association with metastatic disease than those without [4]–[7]. Based on our current understanding of the HGF/c-Met pathway in HCC, several strategies have been proposed for inhibiting HGF and/or c-Met expression or activity at different levels in HCC management [8]–[10]. Although over-expression of c-Met was found to be involved in aggressive liver tumors and associated with poor prognosis in HCC [4], [5], [11], a contradictory deficiency of c-Met in hepatocytes has been reported to initiate tumorigenesis in liver [12].

Recently, the c-Met receptor tyrosine kinase was reported as a potential molecular target for the personalized treatment of HCC in patients with an active HGF/c-Met pathway [4]. HGF-induced activation of c-Met mediates cellular motility and invasion, and is important for cell proliferation, morphogenesis, angiogenesis, as well as protection from apoptosis. Acting as HGF co-receptors, heparan sulfate proteogylcans (HSPGs), heparin and dermatan sulfate (DS) also have a crucial role in the efficient activation of c-Met through ligand oligomerization [13]–[15]. Furthermore, HS and heparin facilitates c-Met signaling and mediates distinct HGF responses such as mitogenesis, cellular motility and invasion in target cells [16], [17]. Heparin also has been used for the prevention or treatment of cancer-associated thrombosis, and positive effects of heparin on patient survival have been reported in several studies [18]. Recently, anticancer and anti-metastatic activities of heparin were described in animal models and in patients with metastatic disease [19]. In contrast to these results, a recent randomized trial showed no overall survival benefit for patients treated with heparin [20], [21]. Despite this single apparent inconsistency, the activities and underlying molecular mechanisms of heparin and HSPGs as potential anti-cancer and anti-metastic agents warrant further investigation. Since the involvement of HGF/c-Met signaling in the invasiveness and metastasis of HCC is known, and heparin mediates HGF-induced biological responses, we hypothesized that heparin is an important regulator of the HGF-induced activities of HCC cells. To test the hypothesis, we first determined the effects of heparin on HGF-induced cellular responses of HCC cell lines. We show for the first time that heparin inhibits HGF-induced adhesion, motility and invasion of HCC cell lines. To understand the underlying molecular mechanisms of these effects, we examined c-Met and downstream signaling molecules by immunoblotting and found that heparin inhibited HGF-induced c-Met phosphorylation and the activation of downstream signaling molecules. We identified differentially expressed genes by cDNA microarray and selected a subset for validation experiments; among these was Egr1. Egr1 is a member of the zinc-finger transcription factor family that regulates wide spectrum of cellular responses from survival to apoptosis, growth to cell cycle arrest and senescence, and differentiation to transformation [22]–[25]. It has been reported that HGF induces Egr1 transcription in HCC cell lines, and that Egr1 regulates HGF-induced biological responses including angiogenesis, scattering, invasion and motility in a variety of cell types [10], [26]–[28]. To test the hypothesis that Egr1 is a critical mediator of inhibition of HGF signaling by heparin, we characterized the expression of Egr1 in HGF and/or heparin treated HCC cells. We found that HGF-induced activation of c-Met signaling increased the expression level of Egr1, parallel to increased adhesion, motility and invasion of HCC cell lines. Heparin, in contrast, inhibited HGF/c-Met signaling and HGF-induced cellular responses via the inhibition of Egr1 expression as well as downstream targets of Egr1 such as Membrane type 1 metalloprotease (MT1-MMP), matrix metallopeptidase 2 (MMP-2) and matrix metallopeptidase 9 (MMP-9).

## Results

### Heparin inhibits HGF-induced, invasion and migration of Hepatocellular Carcinoma (HCC) Cell Lines

**Figure 1:**
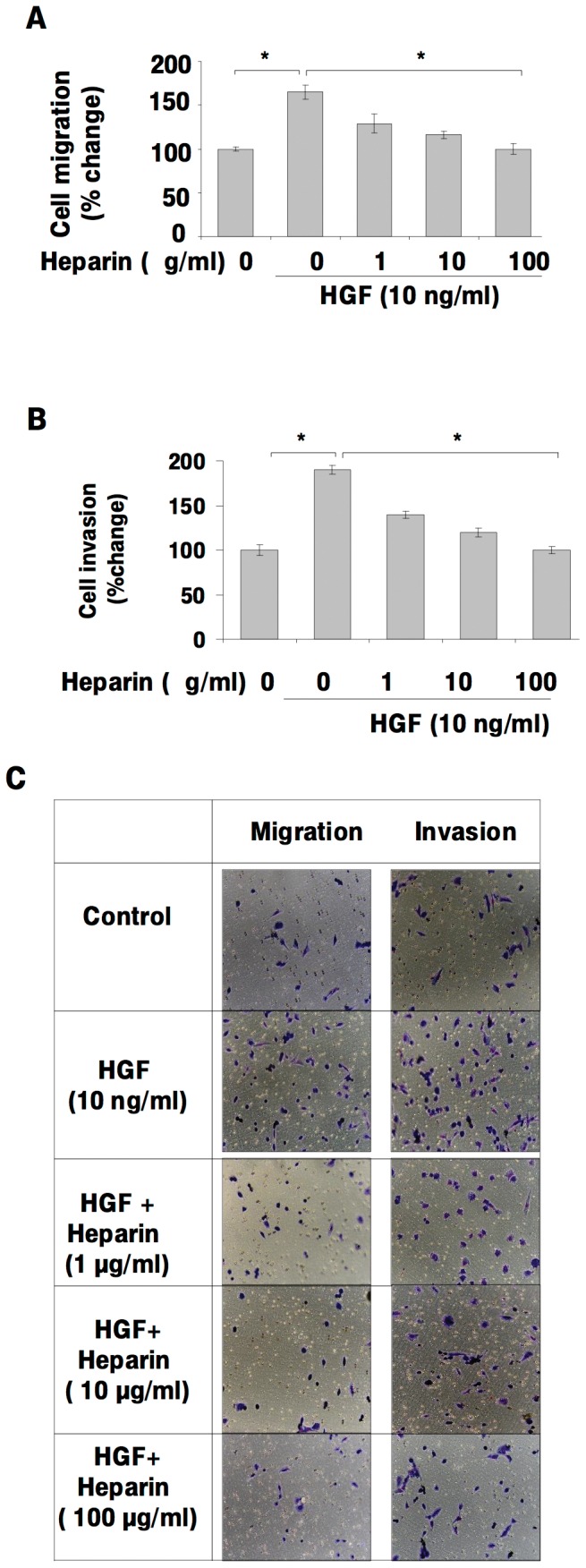
Heparin inhibits HGF-induced cell migration, invasion in the hepatocellular carcinoma-derived cell line SK-HEP-1. The effect of heparin on HGF-induced migration and invasion were analyzed by using modified Boyden chambers and the Roche xCELLigence System in SK-HEP-1 cells. Following serum deprivation, SK-HEP-1 cells were left untreated (0) or treated with HGF in the presence or absence of heparin, then invasion and migration assays were performed. In both panels, values are expressed as the ratio of migrating or invading cells in HGF and/or heparin-treated wells to control wells treated with heparin alone at the indicated concentrations (**1A, 1B and 1C**). Results are representative of three or more independent experiments performed in triplicate. Bars indicate standard error of the mean (SEM), asterisks (*) indicate statistically significant differences between the indicated groups.

To study the role of heparin on HGF/c-Met signaling in HCC, we first analyzed the effects of heparin and/or HGF on the migration and invasion of SK-HEP-1 HCC cells. In the absence of heparin, HGF significantly increased the motility and invasion of SK-HEP-1 cells (p<0.05). However, in the presence of heparin, HGF-induced motility and invasion were significantly reduced in a dose-dependent manner (p<0.05) (Figure 1A, 1B and 1C). Similarly, heparin significantly inhibited (p<0.05) HGF-induced adhesion, motility and invasion by HuH-7 and Hep3B HCC cells (Figure S1A, S1B and S1C). Similar data were obtained with Mahlavu and SNU-449 HCC cells (data not shown). HGF or HGF plus heparin treatment did not significantly affect the proliferation of the HCC cell lines that we have tested (data not shown). Apoptosis was similarly not effected by HGF and/or heparin treatment up to 72 h in SK-HEP-1 cells (data not shown).

### Heparin inhibits HGF-induced expression and transcriptional activity of Egr1

**Figure 2:**
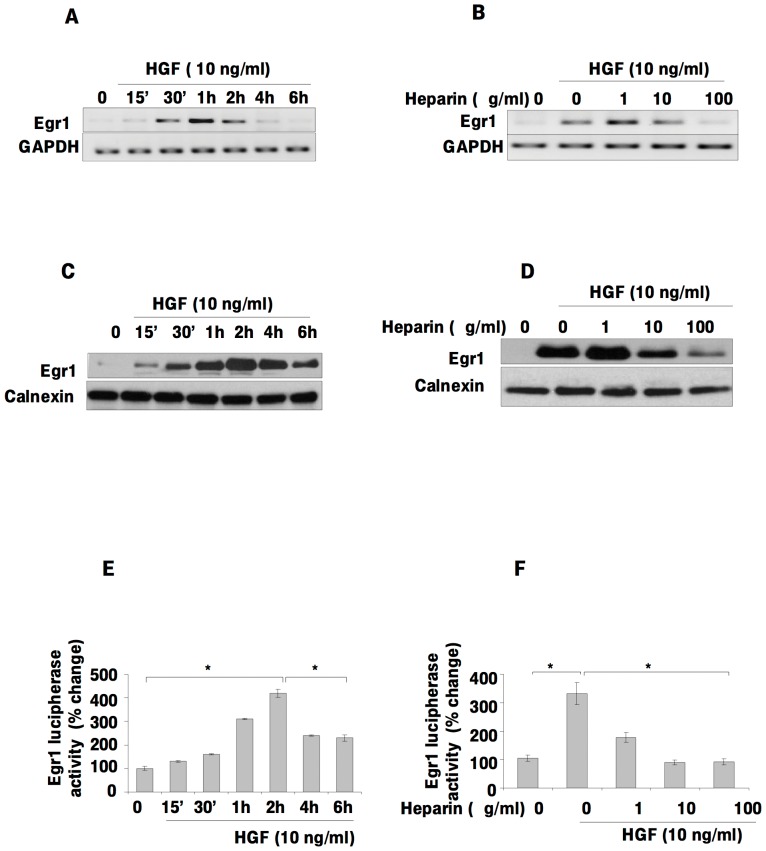
Heparin inhibits HGF-induced Egr1 expression and the Egr1 promoter activity in a dose-dependent manner. HGF-induced activation of Egr1 mRNA and protein level (**2A, 2C**) and the effect of heparin on the HGF induced Egr1 expression in SK-HEP-1 cells were examined by RT-PCR and western blotting (**2B, 2D**). Egr1 transcriptional activity was measured by luciferase reporter assay after transient transfection of SK-HEP-1 with pGL2-Luc-B-Egr1 plasmid construct and the pRL-TK *Renilia* luciferase. Relative luciferase activity was determined as described in Materials and Methods. The firefly luciferase activity was normalized to *Renilia* luciferase activity (**2E, 2F**). Results are representative of two independent experiments performed in triplicate. Bars indicate standard error of the mean (SEM), asterisks (*) indicate statistically significant differences between the indicated groups.

To identify differentially expressed genes in SK-HEP-1 cells treated with HGF and/or heparin, we used the Affymetrix GeneChip cDNA microarray. One of the genes exhibiting a significant difference was Egr1. To confirm that the expression level of Egr1 was affected by HGF and/or heparin treatment, we performed RT-PCR and immunoblot analysis in SK-HEP-1 cells. We observed significantly higher Egr1 mRNA and protein levels in HGF-treated cells compared to untreated cells in a time-dependent manner (Figure 2A and 2C). Heparin treatment led to dose-dependent decreases in the levels of Egr1 mRNA and protein (Figure 2B and 2D). To further investigate the effects of HGF and/or heparin on Egr1 transcription, cells were transfected with pGL2-Luc-B-Egr1 plasmid and luciferase activity was measured. As shown in Figure 2E, HGF induction transactivated the Egr1 promoter in a time-dependent manner. Furthermore, the HGF-stimulated increase in the reporter activity of Egr1 was abolished by heparin treatment in a dose-dependent manner (Figure 2F), consistent with the mRNA results.

### Heparin inhibits HGF-induced activation of c-Met and ERK1/2, MMP-2 and MMP-9

**Figure 3:**
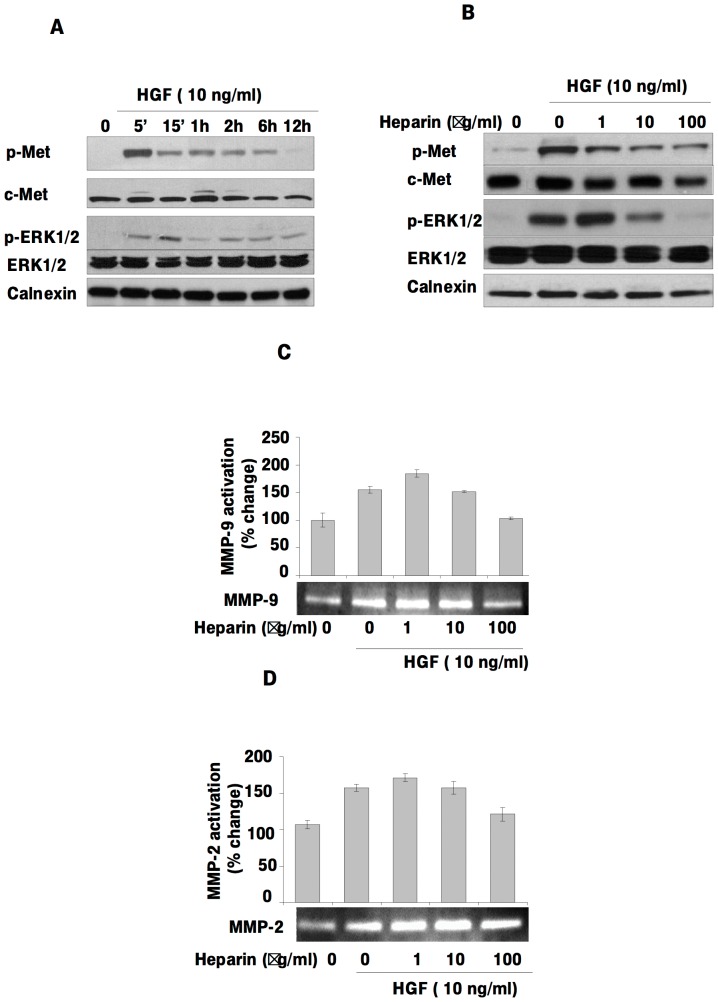
Heparin inhibits HGF-induced p-Met, p-MAPK, MMP-2 and MMP-9 activation in SK-HEP-1. In order to examine the time dependent effects of HGF, overnight starved SK-HEP-1 cells were grown in presence or absence of HGF for the time periods indicated. Total cell lysates were then analyzed by immunoblotting. Blots were probed with anti-p-Met, anti-c-Met, anti-pERK1/2, anti-ERK1/2 and anti-calnexin antibodies. From top to bottom: HGF stimulated c-Met phosphorylation; the amounts of c-Met protein present whole cell lysates; HGF induced ERK1/2 phosphorylation and ERK1/2 and c-Met total protein levels. Membranes were re-probed with ERK1/2 and c-Met antibody after stripping. The bottom panel verifies equal protein loading among the lanes (**3A**). SK-HEP1 cells left untreated or treated with HGF for 2 hours in the presence and absence of heparin. Total protein lysates were analyzed by immunoblotting. Membranes were blotted with anti-p-Met, anti-c-Met, anti-pERK1/2, anti-ERK1/2 antibodies, and anti-calnexin antibodies. The first panel shows the level of tyrosine phosphorylated c-Met; the amount of c-Met protein present in the each lane is shown in the second panel. The third and fourth panels show the amount of phosphorylated ERK1/2 level and the level of ERK1/2 protein in the whole cell lysates, respectively. Membranes were re-probed with ERK1/2 and c-Met antibody after stripping. The lower panels show the amount of calnexin as a loading control (**3B**). Zymographic gel showing active MMP-9 and MMP-2 bands in 24 h conditioned medium from cultured SK-HEP-1 cells left untreated or stimulated with HGF and heparin (**3C, 3D**).

If Egr1 is a target of the HGF/c-Met signaling pathway and heparin inhibits HGF-induced migration and invasion via Egr1, then heparin is likely to inhibit HGF-induced c-Met activation and downstream signaling. To assess c-Met and ERK1/2 activation by HGF in SK-HEP-1 cells, serum-deprived cells were treated with HGF for various periods and c-Met activation status was measured using phospho-Tyr1234/1235-c-Met- and phospho-Thr202/Tyr204-ERK1/2-specific antibodies. The levels of phosphorylated c-Met and ERK1/2 increased in a time-dependent manner upon HGF treatment (Figure 3A). Heparin inhibited HGF-activated c-Met and ERK1/2 activation in a dose dependent manner (Figure 3B). Since previous studies have shown that Egr1 mediates MMP-2 and MMP-9 activation and MT1-MMP expression, we determined the effects of HGF and/or heparin on the levels of MMP-9 and MMP-2 activation and by gelatin zymography. Treatment with HGF increased MMP-9 and MMP-2 activation, whereas heparin suppressed these effects (Figure 3C, 3D).

### Heparin reduces binding of HGF to c-Met

**Figure 4:**
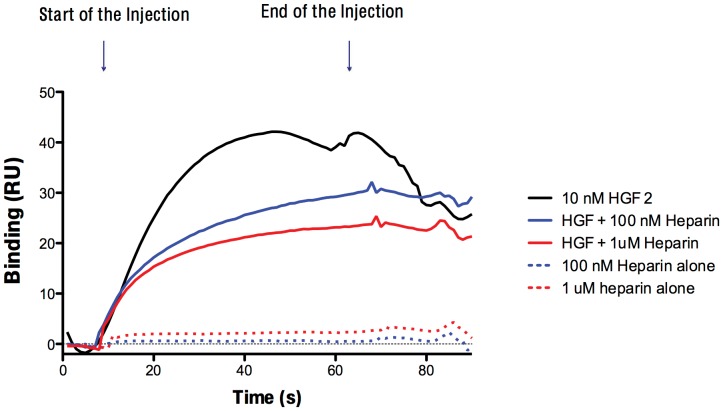
Heparin inhibits HGF/c-Met interaction. HGF binding to c-Met was measured in a Biacore T-100. HGF strongly binds to c-Met (black line), **and the binding decreased upon** heparin treatment in a dose-dependent manner (100 nM Heparin blue line, 1 µM heparin red line). Heparin alone did not show any significant binding to c-Met.

To address the question of whether heparin interferes with binding of HGF to its receptor c-Met, we used surface plasmon resonance spectrometry (Biacore), which directly measures binding in real time (Figure 4). HGF injection over the c-Met coated surface created a specific binding signal. When HGF was premixed with heparin and injected over c-Met surface, dose-dependent inhibition of the HGF/c-Met interaction was observed. When heparin alone was injected, no meaningful binding was detected. Similar data were obtained with four different heparin types.

### The c-Met antagonist SU11274 inhibits Egr1 expression and transcriptional activity

**Figure 5:**
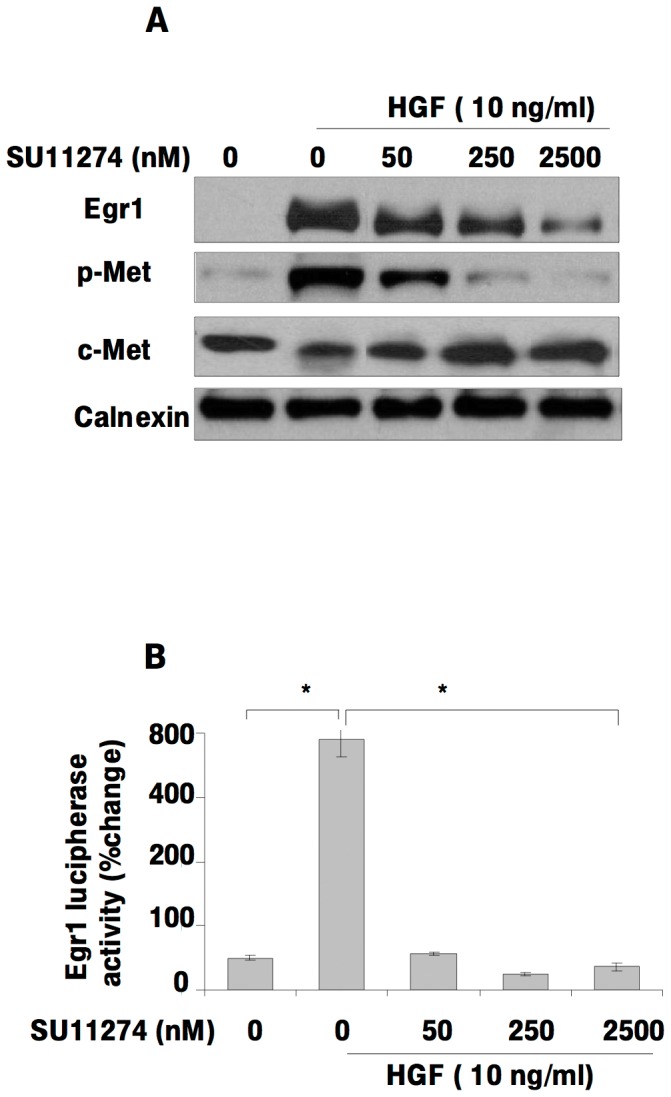
The c-Met inhibitor SU11274 blocks HGF-induced Egr1 expression and Egr1 promoter activity. HGF-induced activation of Egr1 and the effect of c-Met inhibition on HGF-induced Egr1 expression in SK-HEP-1 cells were examined by immunoblotting and luciferase reporter assays (**5A, 5B**). Transcriptional activity of Egr1 was measured by luciferase reporter assay after transient transfection of SK-HEP-1 with a pGL2-Luc-B-Egr1 plasmid construct and pRL-TK *Renilia* luciferase. Relative luciferase activity was determined as described in Materials and Methods. The firefly luciferase activity was normalized to *Renilia* luciferase activity. Results are representative of two independent experiments performed in triplicate. Bars indicate standard error of the mean (SEM), asterisks (*) indicate statistically significant differences between the indicated groups.

We used SU11274, a potent and specific c-Met inhibitor, to further define the role of HGF/c-Met signaling in the regulation of Egr1 expression. Treating SK-HEP-1 cells with HGF in absence and presence of SU11274 showed that HGF-induced p-Met activation and Egr1 expression were significantly decreased by SU11274 (Figure 5A). The HGF-stimulated increase Egr1 reporter activity was abolished by treatment with this c-Met inhibitor in a dose-dependent manner (Figure 5B). These results further support the concept that HGF/c-Met signaling mediates Egr1 activation.

### Egr1 knockdown suppresses HGF-induced invasion by attenuating MMP expression and activation

**Figure 6:**
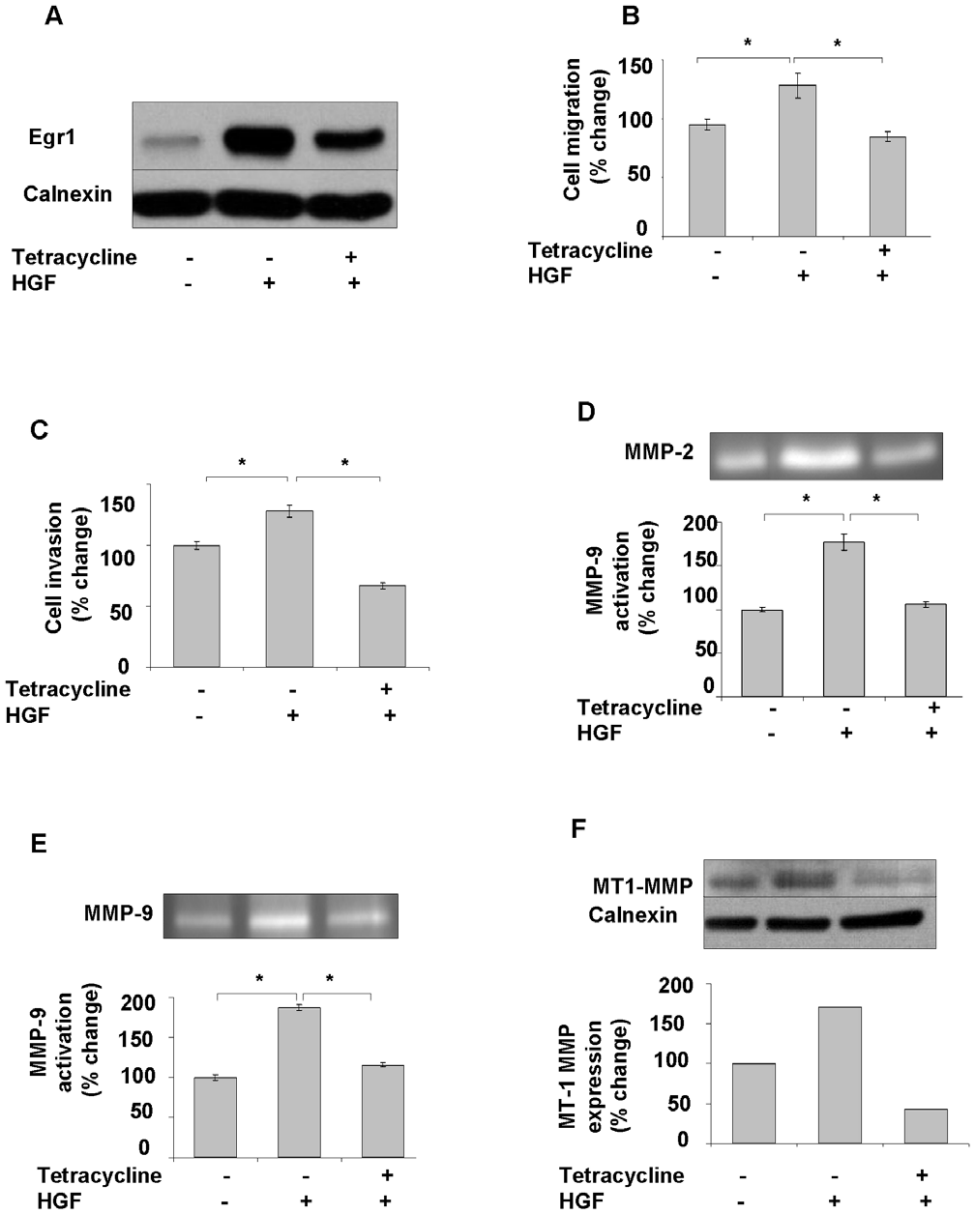
Stable vector-mediated, inducible knockdown of Egr1. To achieve an inducible knockdown of Egr1, we firstly transfected SK-HEP-1 cells with pCDNA6/TR which express tet repressor. Stable cells were selected using Blasticidin before co-transfecting SK-HEP-1 T-REx cells with tetracycline–regulated pSUPER.retro.neo+GFP tet RNAi construct, which was directed against a sequence containing Egr1. Stable cells were selected using G418. After selection, clones were analyzed for downregulation of Egr1 after 24 h of tetracycline treatment. Ten-fold down-regulation of Egr1 was detected by immunoblotting in pSUPER.retro.neo+GFP tet/Egr1 clones (**6A**) but not in mock transfected clones (**6B**). The graph compares the signal intensities obtained from Egr1 bands.

**Figure 7:**
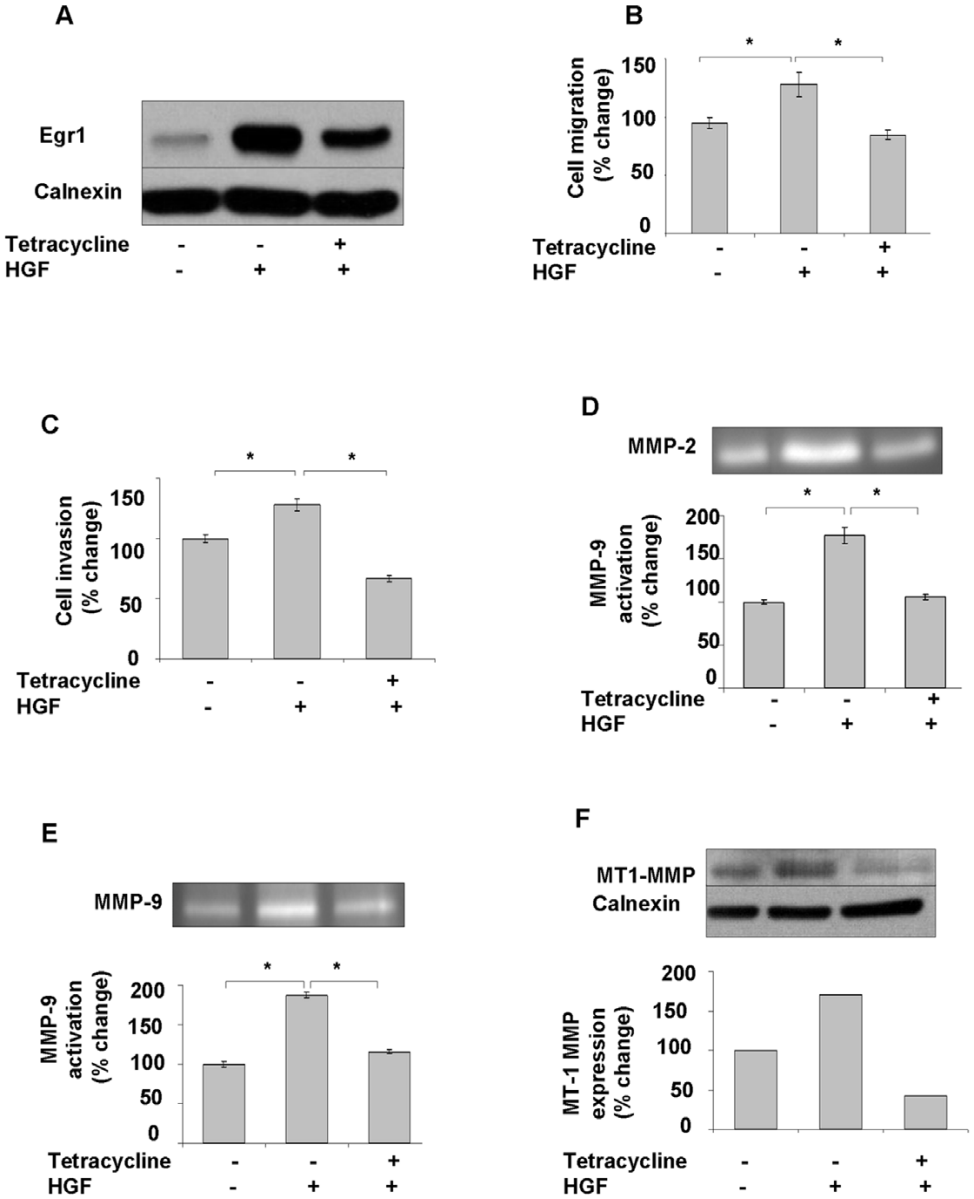
Knockdown of Egr1 decreases HGF-mediated invasion and activation of MMP2, MMP9, and expression of MT1-MMP. SK-HEP-1 TREx cells were obtained by stable transfection of pCDNA-6/TR. Then SK-HEP-1 TREx cells were transfected with recombinant pSUPER-retro.neo+GFP vector expressing Egr-1 targeted shRNA or empty pSUPER-retro.neo+GFP vector using Fugene as described in Materials and Methods. Knockdown of Egr1 in Egr1-shRNA-expressing SK-HEP-1 stable cell lines were induced by tetracycline (**7A**). Equal amount of tetracycline induced and un-induced Egr1-shRNA-expressing SK-HEP-1 cells were used for migration and invasion assays in the presence and absence of HGF (**7B, 7C**). Results are expressed as fold differences of matrigel invaded cells and are representative of two or more independent experiments performed in triplicate. Bars indicate standard error of the mean (SEM), asterisks (*) indicate statistically significant differences between the indicated groups. Zymographic gel showing active MMP-2 and-9 bands in 24 h conditioned medium of cultured Egr1-shRNA-expressing SK-HEP-1 cells left untreated or stimulated with tetracycline and/or HGF (**7D, 7E**). Protein levels of MT1-MMP were studied in tetracyclin and/or HGF-induced SK-HEP-1-Egr1 cells (**7F**). Densitometric analysis of gelatin zymography and immunoblots showed that HGF induces MT1-MMP expression and MMP-2 and -9 activation, whereas silencing of Egr1 blocks HGF-induced MMPs up-regulation.

**Figure 8:**
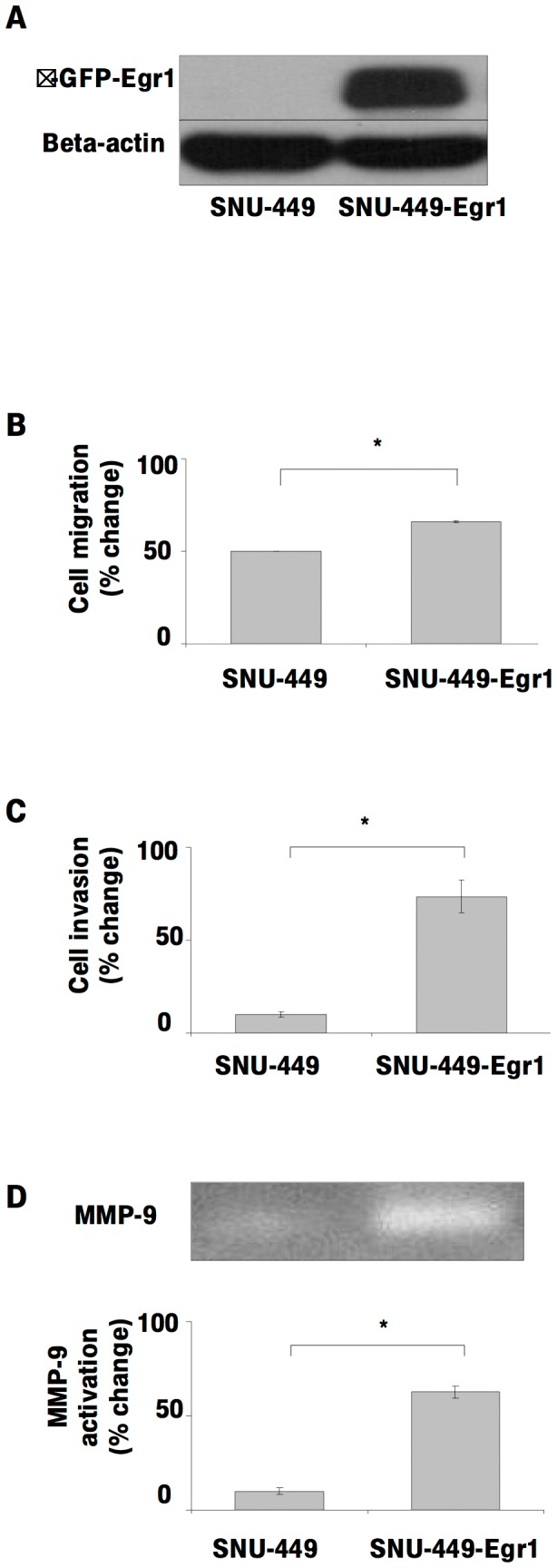
Overexpression of Egr1 induces MMP-9 activation, cell invasion and cell migration of HCC cells. The effect of Egr1 on the cell invasion of HCC cell lines were examined using SNU-449 transiently transfected with pCMV-6-AC-GFP Egr1 overexpression vector (**8A**). Equal amounts of wild type and Egr1 overexpressing SNU-449 cells were used for migration (**8B**) and invasion (**8C**) assay in the presence and absence of HGF. Results are expressed as fold differences of matrigel invaded cells. Zymographic gel showing the active MMP-9 bands in 24 h conditioned medium of cultured Egr1-overexpressing SNU-449 cells (**8D**). The graph compares the signal intensities obtained from zymography gels.

**Figure 9:**
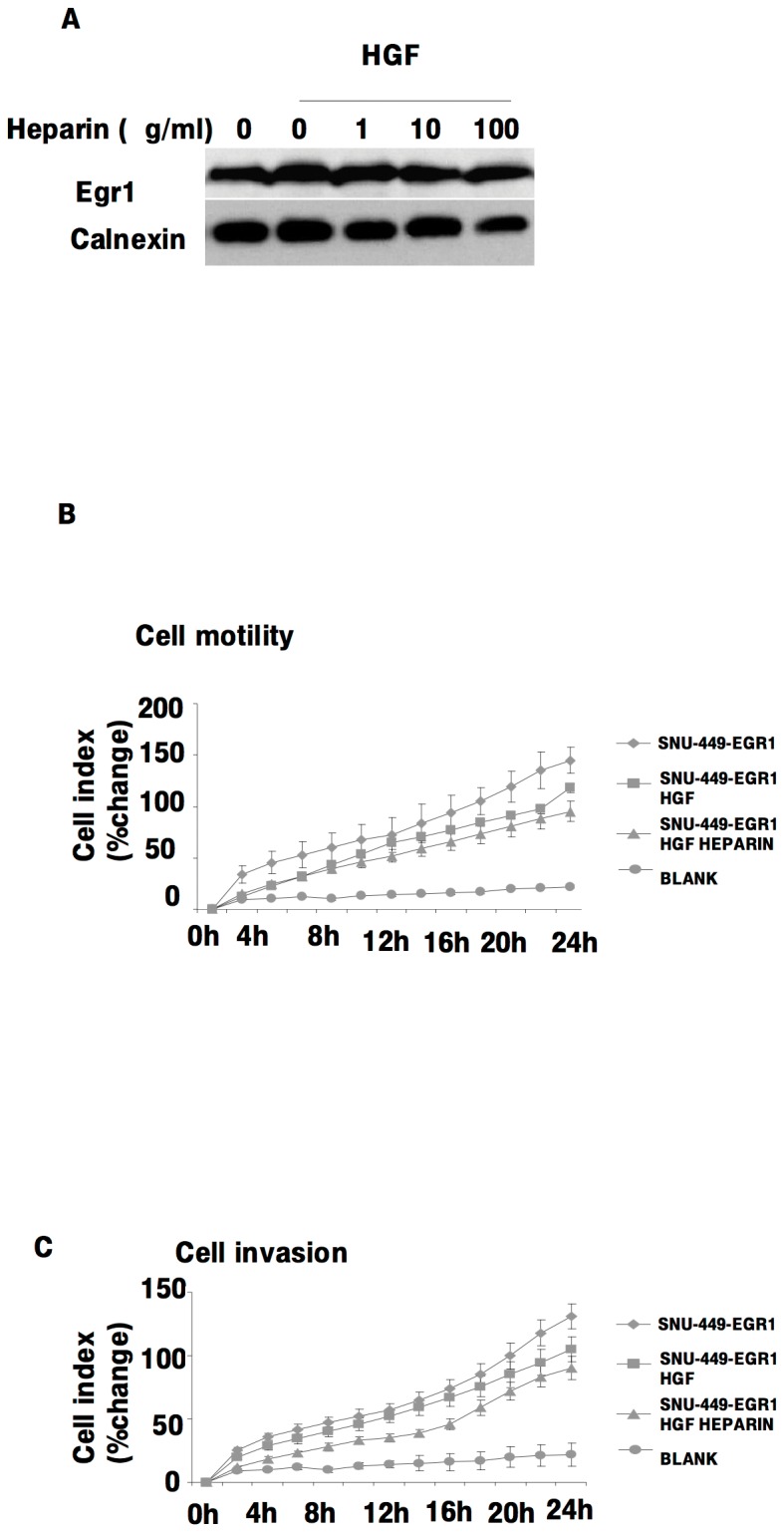
Heparin and HGF do not effects Egr1 expression, motility or invasion in Egr1 over-expressed SNU-449 cell lines. The effects of heparin and HGF on Egr1 expression in Egr1 over-expressing SNU-449 cells was analyzed by immunoblotting (**9A**). Following serum deprivation, Egr1 overexpressed SN-449 cells were left untreated (0) or treated with HGF in the presence or absence of heparin, then invasion and migration assays were performed using the Roche xCELLigence System (**9B, 9C**). Results are representative of three or more independent experiments performed in triplicate. Bars indicate standard error of the mean (SEM), asterisks (*) indicate statistically significant differences between the indicated groups.

To investigate the direct involvement of Egr1 in HGF-induced cell invasion, we first transfected SK-HEP-1 cells with pCDNA 6/TR which express that tet repressor. Stable cells were co-transfected with the tetracycline–regulated pSUPER.retro.neo GFP tet RNAi construct, which was directed against a sequence containing Egr1. Stable cells were then analyzed for downregulation of Egr1 after 24 h of tetracycline treatment. Ten-fold down-regulation of Egr1 was detected by immunoblotting in pSUPER.retro.neo+GFP tet/Egr1 clones (Figure 6A) but not in mock transfectants (Figure 6B). Egr1 knockdown resulted in a significant decrease of HGF-induced cell migration and invasion (p<0.05) (Figure 7A, 7B, 7C). Whereas HGF induced the activation of MMP-2 and MMP-9, and the expression of MT1-MMP, Egr1 knockdown resulted in decreased activation and/or expression of these MMPs (Figure 7D, 7E, 7F), indicating that Egr1 is required for HGF-mediated MMP-2 and MMP-9 activation and MT1-MMP expression. Similar data were observed with stable knockdown of Egr1 in Hep3B cells (Figure S2). To further demonstrate the critical role of Egr1 in HCC cell invasion and motility, we transiently transfected SNU-449 cells with the pCMV6-AC-GFP-EGR1 plasmid to generate Egr1 overexpression. Constitutive overexpression of Egr1 (Figure 8A) resulted in increased basal (unstimulated) motility (Figure 8B) and an 8-fold increase in basal invasion of SNU-449-Egr1 cells (Figure 8C). Consistent with the observed increased in invasiveness, Egr1 overexpression induced MMP-9 proteolytic activity (Figure 8D). These cells did not express MMP-2 or MT1-MMP, thus the effects of Egr1 on their expression and/or activity could not be determined. As anticipated, treatment of SNU 449-pCMV6-AC-GFP-EGR1 cells with HGF or HGF plus heparin did not further increase Egr1 expression (Figure 9A) or cell motility and invasion (Figure 9B, 9C).

## Discussion

HGF/c-Met signaling regulates cellular motility, invasion, and metastasis in various types of human cancer [29], [30]. It has been reported that activation of HGF/c-Met signaling is associated with intrahepatic metastasis, vascular invasion, poor prognosis, and drug resistance in HCC. Therefore, the c-Met receptor tyrosine kinase is thought to be a promising target for the personalized treatment of HCC [1], [30], [8]. Recent studies also suggest that due to its anti-metastatic and anti-invasive activities, heparin may be a potential therapeutic for the treatment of cancer [20], [21]. Although there is considerable information on the role of heparin in HGF/c-Met signaling generally, the effects of heparin on HGF/c-Met signaling in HCC are not yet known.

We show for the first time that heparin inhibits HGF-induced cell invasion, cell motility, and cell adhesion in HuH-7, Hep3B, SK-HEP-1, Mahlavu HCC cell lines. The effects of heparin on HGF-induced migration by other cell types have been reported in a few studies. Rubin et al [16] reported that heparin facilitates HGF signaling through interaction with c-Met and the N domain of HGF, and heparin treatment increases both mitogenesis and motility in HS deficient 32D/c-Met cells. Similarly, Lyon et al. [15] showed that the activity HGF was dependent on the presence of sulfated GAGs on the cell surface and that both HS and DS increased HGF induced cell motility in GAG deficient CHO pgsA-745 cells. Most, if not all HGF target cells possess abundant cell surface HS and DS proteo- and glycosaminoglycans. Thus, these studies identify an important positive role for cell surface HS and DS proteoglycans in HGF signaling, and suggest that soluble heparin may competitively antagonize the effects of these cell surface glycans, consistent with the results as we present here for HCC cells. Surface plasmon resonance results also supported the hypothesis that added soluble heparin can inhibit the interaction of HGF with c-Met.

HGF-induced motility and invasion has become an important target for the prevention and/or treatment of metastasis in HCC. The stimulatory effects of HGF on the motility and invasion of HCC cells are well documented [1], [5], [31], [32], and activation of cell motility and invasion are also rate-limiting steps of tumor metastasis [33]. Concordantly, the activation of HGF/c-Met signaling positively correlates with tumor metastasis in HCC [1]. In addition, HGF/c-Met signaling induces epithelial-mesenchymal-transition (EMT), a pivotal event in the development of invasive and metastatic cancer progression in HCC cells [34]. We propose that heparin may be an effective inhibitor of motility, invasion and metastasis in this context. In support of this concept, we note that positive effects of heparin on the survival of cancer patients are documented in animal models and clinical studies [21], [35]. In 2011, Akl et al. [35] conducted a systematic review on the effects of heparin on the survival of cancer patients with no therapeutic or prophylactic indication for anticoagulation and reported a potential survival benefit from heparin therapy. Moreover, the randomized controlled trials that were reviewed did not report any significant effects on bleeding, suggesting that the survival advantage of heparin might be related to its interaction with signaling molecules. Recent reports suggest that the therapeutic effects of heparin for HCC and other cancers also might be due to inhibition of cell invasion and metastasis, rather than inhibiting the growth of primary tumors [36]–[38]. Consistent with those studies, in the presence HGF we did not observe significant effects of heparin on cell proliferation; HGF treatment alone did not induce the proliferation of HCC cells that were studied up to 96 hrs. We note that although the effects of HGF on the proliferation of primary hepatocytes are well documented, the role of HGF on the proliferation of HCC cells remains controversial [39], [40]. Stimulatory effects of HGF on the proliferation of some HCC cell lines have been reported [39], whereas the anti-mitotic and apoptotic effects were reported by others [40].

In order to identify genes that regulate HGF-stimulated migration and invasion and that were also sensitive to the inhibitory effects of heparin, we used cDNA microarray expression profiling. We provide evidence that Egr1, a zinc finger transcription factor known to mediate HGF-induced motility and invasion, had significantly reduced activity as a regulator of HCC cell invasion in the presence of heparin. The diverse involvement of Egr1 in the regulation of growth, differentiation, survival and stress responses has been reported in various models [22], [41], [42]. Egr1 protects normal cells from transformation by inducing apoptosis or growth arrest upon DNA damage, and its role in chemoresistance and radioresistance in tumor cells is well documented [43]–[45]. In breast, colon and lung cancer, fibrosarcoma and glioblastoma, Egr1 is considered a tumor suppressor gene [46]–[48], but Egr1 can act both as a tumor suppressor and as a tumor promoter, depending on the cell context [22], [49].

While Egr1 is crucial for the proliferation of hepatocytes and plays an important role in liver regeneration [50], Egr1 expression level and its impact in HCC are controversial. One study reported Egr1 overexpression in HCC [51], whereas another found downregulation of Egr1 expression [52]. Recent data suggests that different biological functions of Egr1 in HCC might arise due to increased HGF/c-Met signaling [25], [26]. Various studies show that Egr1 is a critical transcription factor in mediating HGF-induced expression of genes that encode regulators of cell migration, invasion, angiogenesis, malignant progression and metastasis [25]–[27], [53]. We show that HGF induction of MMP-2 and MMP-9 activation, and MT1-MMP expression, is also mediated by Egr1 in HCC. The only other known mediator of HGF induced transcription of MMPs was Ets-1 [54]. Egr1 mediation of MMP activation and expression by HGF is critical in the context of HCC progression because several studies have shown that in human HCC, these MMPs are associated with metastasis and aggressive behavior [55], [56]. We further provide functional evidence that Egr1 is a critical regulator of HGF-induced invasion by HCC cells: silencing Egr1 in SK-HEP-1 and Hep3B HCC cell lines decreased HGF-induced invasion and MMP secretion, and Egr1 overexpression in SNU-449 cells increased invasion and MMP levels. Our results predict that the impact of HGF-induced Egr1 activity in HCC is strongly pro-metastatic.

We show that heparin inhibited HGF-induced Egr1 expression and gene promoter activity in a dose- and time-dependent manner, providing a basis for inhibition of HGF-induced activation of MMP-2 and 9 through decreased MT1-MMP expression. Consistent with this mechanism, the silencing of Egr1 abolished the inhibitory effects of heparin on HCC cell invasion. Heparin inhibition was a direct consequence of decreased HGF-induced c-Met phosphorylation and pERK1/2 activation. The use of specific inhibitors of c-Met and MAPK confirmed that HGF-induced Egr1 expression and cell invasion depend on the MAPK pathway in HCC, as reported previously [22], [26], [57]. Consistent with these findings, the ectopic overexpression of Egr1 in HCC cells rescued cells from the inhibitory effects of heparin on HGF-induced invasion.

**Figure 10:**
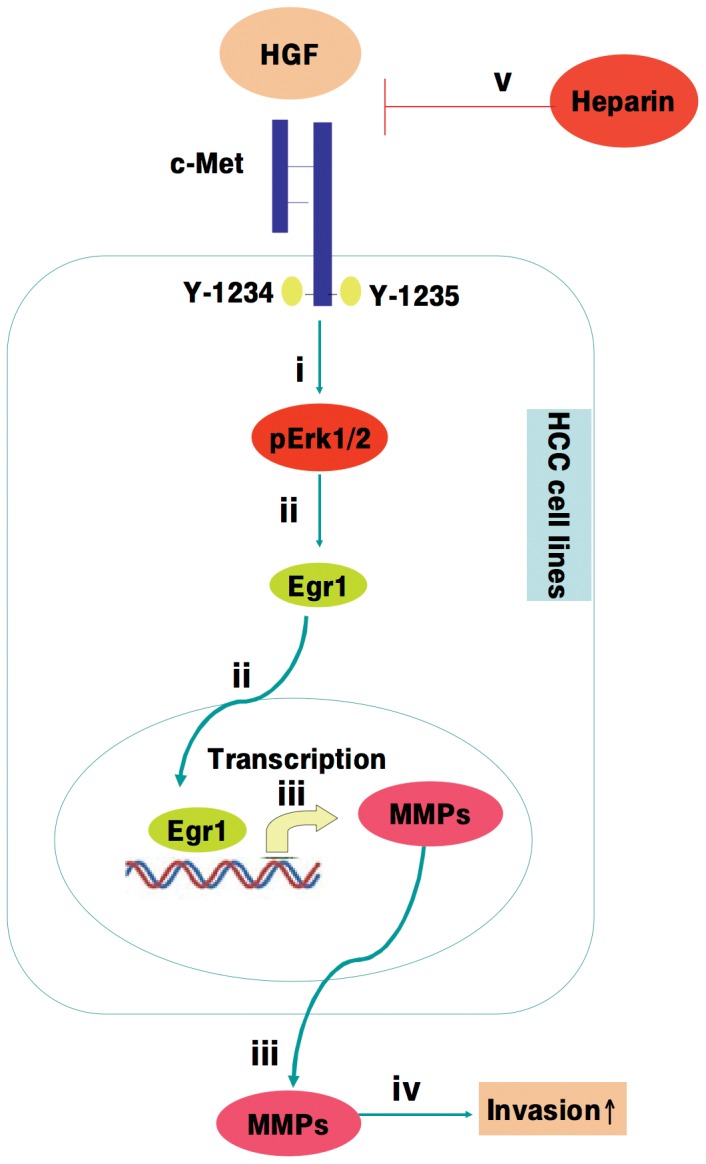
The mechanism of heparin inhibition of HGF-induced cell motility and invasion. Activation of HGF/c-Met signaling in HCC cells leads to MAPK signaling (i), and up-regulation of Egr1 (ii), which in turn induces MMP-2 and MMP-9 activation and MT1-MMP expression (iii), and increased cell motility and invasion (iv). Heparin treatment results in the inhibition of HGF-induced cellular invasion via repression of HGF-induced c-Met activation, as well as inhibition of MAPK signaling, downregulation of Egr1 expression, and MMP activation (v).

Together, our results define the molecular signaling pathway by which heparin inhibits the HGF-induced expression of transcription factor Egr1 and in turn, HCC cell motility and invasion (Figure 10). These studies provide functional and mechanistic support for further preclinical and clinical investigations into the use of heparin to prevent and treat HCC metastastic progression.

## Materials and Methods

### Reagents

All cell culture supplies were from Biological Industries (Israel). HGF was from R&D Systems (MN, USA). Heparin was from Calbiochem 375093 (USA). Anti-Egr1 (15F7) cs 4351, Anti-phosho-Met (Y-1234/1235) cs 3129 and anti phosho-44/22 MAPK (Thr202/Tyr204) (19G2) 4377 antibodies were from Cell Signaling Technology (Danvers, MA, USA). Anti-Met (C-28) sc-161, anti-ERK1 (C-16) sc-93, MT1-MMP (H-72) sc-30074 anti-Calnexin (H-70) sc-11397 antibodies were from Santa Cruz Biotechnology (Santa Cruz, CA USA). HRP-anti-mouse/rabbit secondary antibodies were from Pierce (IL, USA). ECL detection system was from Pierce (II, USA). c-Met inhibitor SU11274 was from Calbiochem 448101 (Darmstadt, Germany). Tetracycline was purchased from Sigma C4881.

### Cell Culture

Human hepatocellular carcinoma cell lines HuH-7, Hep3B, SNU-449, SK-HEP-1, and Mahlavu were cultured in DMEM (Dulbecco's modification of Eagle's) medium supplemented with 10% FBS (Fetal Bovine Serum) 100 U/ml penicillin, 0.1 mg/ml streptomycin, 2 mM L-glutamine and 1% MEM, (Minimum Essential Medium) Non-Essential Amino Acids solution in a humidified 5% CO_2_ incubator at 37°C. HCC cell lines were kindly provided by Dr. Mehmet Öztürk [58]. HGF (10 ng/ml), heparin (0, 1, 10, 100 µg/ml) were used together at specific time points after overnight incubation in DMEM with 2% FBS. For the inhibition of c-Met, SU11274 (0, 50, 250, 2500 nM) (Calbiochem 448101) were added to the cultures with HGF at specific time points. DMSO was used as solvent control for SU11274, which is dissolved in DMSO (Applichem). For the silencing of Egr1, Egr1 knockdown SK-HEP-1 cells were treated with 1 µg/ml tetracycline for 24 h.

### *In-vitro* motility and invasion assays

*In-vitro* motility and invasion assays were performed as described [59]. The motility and invasion abilities of SK-HEP-1, Egr1 overexpressed-SNU-449 and Egr1 knockdown-SK-HEP-1 Egr1.1.16 were measured using 8 µm pore size of Biocat Cell Enviroment control and matrigel invasion inserts, respectively (BD Bioscience, CA, USA). Briefly, %70 confluent cells were starved overnight with DMEM containing 2% FBS and cell line were treatment with tetracycline (1 µg/ml) in DMEM containing 2% FBS for 24 hrs. Untreated cells were used as a control. Lower chambers were filled with 750 µl of DMEM containing 2% FBS, to which HGF (40 ng/ml) and/or heparin and tetracycline were added at the indicated concentration. Cells were trypsinized, washed in DMEM and added to upper chambers (1×10^4^ cells/insert) with heparin and tetracycline at the indicated concentrations and incubated at 37°C for 24 h. After incubation, media was removed, cells on the upper portion of each membrane were wiped with cotton swabs, whereas cells that had transversed through the membrane were fixed and stained with Diff-Quick (Siemens Healthcare Diagnostics, IL, USA). Total cell numbers on the bottom surface of the membranes were counted using brightfield microscopy. Three or more independent experiments were performed in at least triplicate. Bars represent fold difference in mean migrating or invading cell numbers. Fold differences were calculated by dividing the experimental results by the control results. Invasion and motility of cells were also monitored using a real-time cell electronic sensing RT-CES system (xCeLLigence-Roche Aplied Science [60]. For real time motility analysis, the lower chambers filled with DMEM containing 2% FBS, to which HGF (40 ng/ml) and/or heparin were added at the indicated concentrations. Measurements of cell index were taken every 30 min. For invasion studies, the membrane on the bottom of the top chamber of CIM plates were coated with 25 µL of a 0.25 mg/ml of Matrigel (BD Biosciences) in serum free media and incubated at 37°C for 2 hours. Then 2×10^4^ cells were added to the upper chamber in DMEM with 2%FBS, which contained heparin and tetracycline at the indicated concentrations. Subsequent steps were performed in the same manner as described for cell migration assays.

### Reverse Transcriptase PCR (RT-PCR)

SK-HEP-1 cells were cultured until 60%–70% confluence and serum starved overnight. Cells were then treated with HGF and/or heparin at the indicated time points. Primers for Egr1 gene (F5′CTCTCTGAACAACGAGAAGGTGCT3′ R5′AGATGGTGCTGAGGA CGAGGA) were selected with aid of PrimerQuest software (Integrated DNA Technology). Glyceraldehyde-3- phosphate dehydrogenase (GAPDH) was used as an internal control. Total RNA was prepared using Trizol, and reverse-trancripted into c-DNA by using M-MuLV Reverse Transcriptase kit (MBI Fermentas, USA). Synthesized c-DNAs were used as template for PCR (Polymerase Chain Reaction) reactions. All PCR reactions were carried out using 2 µl/cDNA from the reverse transcription mix which was performed using 2 µg total RNA, for 28 cycles. Amplified products were analyzed by electrophoresis on 2% (w/v) agarose gels. Negative controls without reverse transcriptase and without DNA were included for each set of experiments. The gels were photographed using a transilluminator system (Vilber Lourmat). All RT-PCR supplies were from Fermentas, MBI (USA). Band intensities were quantified as pixels by using ImageJ software.

### Luciferase Reporter Assay

The pGL2-Luc-B-Egr1 luciferase reporter construct was kindly provided by Dr. Işıl Kurnaz (Yeditepe University, Ankara, Turkey) with permission of Stephen Safe (USA) [61]. For reporter gene analysis, SK-HEP-1 cells were seeded at 25.000 cells/per well in a 12 well plate for 24 hours before transfection. Cells were then transfected with the pGL2-Luc-B-Egr1 plasmid construct using Fugene HD Transfection Reagents Roche (Mannheim, Germany) according to manusfacturer's instructions. For normalization, pRL-TK *Renila* luciferase reporter was co-transfected. After transfection, cells were starved overnight then treated with heparin (1, 10, 100 µg/ml Calbiochem), c-Met inhibitor SU11274 (0, 50, 250, 2500 nM) and HGF (10 ng/ml, M110) for 1 hour. Cells were harvested in luciferase lysis buffer and the luciferase activity was analyzed in each cell lysates using Dual-Glo Luciferase Assay System (Promega, Madison, WI, USA) with a luminometer (Turner Designs 20/20^n^ Sunnywale, CA). Relative luciferase unit (RLU) represents firefly luciferase normalized against Renilia luciferase activity.

### Short Hairpin RNA (shRNA) mediated Egr1 knockdown

A 19-base pair (bp) oligonucleotide targeted against nucleotides 152–171 of the Egr1 mRNA (5-GAGGCCTTCTGGATTGACA-3) was designed using the workstation software from OligoEngine. The 64-nucleotide short hairpin RNA sense primer 5- GATCCCCGAGGCCTTCTGGATTGACATTCAAGAGATGTCAATCCAGAAGGCCTCTTTTTGGAAA-3 and the antisense primer 5-AGCTTTTCCAAAAAGAGGCCTTCTGGA TTGACATCTCTTGAATGTCAATCCAGAAGGCCTCGGG-3 were annealed and ligated into the modified pSUPER.retro.neo GFP tet vector (OligoEngine) according to the manufacturer's instructions. To obtain SK-HEP-1 TREx cells, pCDNA 6/TR (Invitrogen) were transfected into SK-HEP-1 using FUGENE HD reagent (Roche Diagnostic) according to the manufacturer's instructions. Stable transfected cells were selected with 6 µg/ml Blasticidin S (Invitrogen). To generate shRNA SK-HEP-1 T-Rex (Tetracycline-Regulated Expression) cells, pSUPER.retro.neo GFP+tet/Egr1 was co-transfected and stable cell lines were selected using 1000 µg/ml G418 (Roche Diagnostic). Knockdown of Egr1 in SK-HEP-1 Egr1.1.6 cell lines was induced by addition of 1 µg/ml tetracyclin treatment for 24 h. SK-HEP-1 TREx cells transfected with an empty vector (mock) were used as a negative control.

### Immunoblotting

Total cell lysates were prepared from SK-HEP-1 and SK-HEP-1 Egr1.1.16 cell lines with RIPA buffer (50 mM Tris-CI pH 7.4, 150 mM NaCI, 1 mM EDTA pH 8.0, %1 NP-40, 1× protease inhibitor cocktail, 1 mM NaF, 1 mM Na_3_VO_4_). Protein concentrations were determined by BCA assay according to the manufacturer's instructions (Pierce, IL, USA). Equal volumes of lysates were loaded onto 10% SDS polyacrylamide gels for electrophoretic analysis. The proteins in the gel were transferred to PVDF membranes (Pierce), which were first blocked with Tris-buffered saline/0.1% Tween-20 (TBST) containing 5% nonfat dry milk for 1 hour at room temperature. Membranes then was blotted with primary antibodies against phospho-Met, Egr1, MT1-MMP, calnexin, phospho ERK1/2, ERK1/2 in TBST containing 3% NFDM, and c-Met (C-28) sc-161 in phosphate buffer saline with 0.1% Tween-20 containing 3% bovine serum albumin overnight at +4°C. Proteins were detected by HRP-conjugated anti-rabbit (Pierce) and anti-mouse secondary antibodies (Pierce), with visualization by the ECL detection system (Pierce). The specific bands were recorded on X-ray film. Equal loading and transfer were confirmed by repeat probing with calnexin. Band intensities were quantified as pixels using ImageJ software.

### Transient Transfection of Egr1 expression vector

SNU-449 cells were transfected with p CMV6-AC-GFP-EGR1 vector (Origene, RG209956, USA), using Fugene HD Transfection Reagents Roche (Mannheim, Germany) according to the manufacturer's instructions. After 48 h cells were harvested and cell lysates normalized for protein concentrations and western-blotting was performed to determine the protein level of Egr1.

### Gelatin-Zymography

The activities of MMP-2 and MMP-9 in conditioned medium were measured by gelatin-zymography assays. Briefly, cells were grown in serum-free medium then treated with HGF (40 ng/ml) and/or heparin (1, 10, 100 µg/ml) for 24 hrs. SK-HEP-1 Egr1.1.16 clone were treated with HGF (40 ng/ml) and tetracyclin (1 µg/ml) for 24 h and cultured supernatant was directly used for detection of secreted MMPs. Cultured cell media were prepared with SDS sample buffer without boiling or reduction, and then equal amounts of medium for each condition were loaded on 10% polyacrylamide gels containing 0.1% SDS and 1 mg/ml gelatin. After electrophoresis the gels were washed with 2.5% Triton X-100 and then incubated in 50 mM Tris-CI (pH7.0) solution containing 10 mM CaCl and 150 mM NaCI at 37°C overnight. Then, the gels were stained with 0.25% Coomassie Brilliant Blue R-250 (Bio-RAD) solution for 1 h and destained with destaining buffer (40% methanol, 20% acetic-acid) until bands became clear. Gelatinolytic activity was visualized as a transparent band against a blue background. Relative densities of MMP-2 and MMP-9 bands were measured by scanning the photographic negatives and quantified as pixels by using ImageJ software.

### Cell Adhesion and Proliferation Assays

Cell proliferation rate was determined using the CyQUANT NF Cell Proliferation Assay (Invitrogen) according to the manusfacturer's protocol. Briefly, SK-HEP-1 cells were seeded in 96-well plates at a density of 1×10^3^ cell/ml. The next day, near-confluent cells were starved overnight in DMEM with 2%FBS. Then cells were treated with HGF (40 ng/ml) and/or heparin (1, 10 and 100 µg/ml) for 24 and 48 hrs. Growth media was later removed, green-fluorescent CyQUANT GR dye was added to the wells and incubated for 30 min at 37°C. The fluorescence intensity of each sample was measured using a fluorescence microplate reader with excitation at 485 nm and emission detection at 530 nm (Biotek, USA). Results represent averages of triplicate samples obtained from at least two independent experiments. Proliferation and adhesion were also monitored using xCeLLigence system (Roche Aplied Science) for 72 h.

### Surface Plasmon Resonance Binding Assays

Experiments were done with a Biacore T-100 instrument and CM5 sensorchips. HBS-P (10 mM HEPES, pH 7.4, 150 mM NaCl and 0.05% P-20) was used as the running buffer with 10 ul/min flow rate. All flow cells were coated with 4000 RU protein A/G using amine coupling method according to manufacturer's instructions (GE Healthcare). IgG-Fc was loaded (100 RU) to flow cell #1 to be used as reference surface. Fc tagged recombinant human c-Met was loaded (350 RU) to flow cell #2. All HGF and heparin injections were done for 60 seconds over both flow cells and the reference-subtracted signal (flow cell #2-#1) was provided in the figures. Raw data was exported to Prism software (v5.0, GraphPad Software, La Jolla, CA) for drawing final figures.

### Statistical analysis

Data were analyzed by Mann-Withney U test. P values of ≤0.05 were considered statistically significant. All statistical procedures were performed using Minitap-15 software.

## Supporting Information

Figure S1 **Heparin inhibits HGF-induced adhesion, migration and invasion in HuH-7 and Hep3B HCC cell lines.** The effect of heparin on HGF-induced migration and invasion of HuH-7 and Hep3B cell lines were analyzed by using Roche xCELLigence System. Briefly, serum deprived HuH-7 and Hep3B cell lines were left untreated or treated with HGF in the presence or absence of heparin. Adhesion, migration and invasion assays were then performed. E-PLATES, CIM-plates and Matrigel coated CIM-plates were used for real time adhesion, motility and invasion assays, respectively. In both panels, values are expressed as the ratio of adhesive (**1A**), migrating (**1B**) or invading (**1C**) cells in HGF and/or heparin-treated wells. Bars indicate standard error of the mean (SEM), asterisks (*) indicate statistically significant differences between the indicated groups.
(TIF)

Figure S2 **Stable knockdown of Egr1 significantly decreases HGF-induced cell invasion in Hep3B cells.** To achieve Egr1 knockdown, Hep3B cells were first transfected with the pSUPER.retro.neo+GFP tet RNAi construct. Stable cells were selected using G418 and marker-selected clones were analyzed for downregulation of Egr1 by western blotting (**2A**). The graph compares the signal intensities obtained from Egr1 bands. HGF-induced cell invasion in the Egr1 knockdown Hep3B cell line was examined using modified Boyden chamber assays (**2B**).
(TIF)
