# Supplementary figures and images for: Heparin Inhibits Hepatocyte Growth Factor Induced Motility and Invasion of Hepatocellular Carcinoma Cells through Early Growth Response Protein 1
Source: PLoS One. 2012 Aug 13;7(8):e42717. doi: 10.1371/journal.pone.0042717 (PMC3418296; doi:10.1371/journal.pone.0042717)

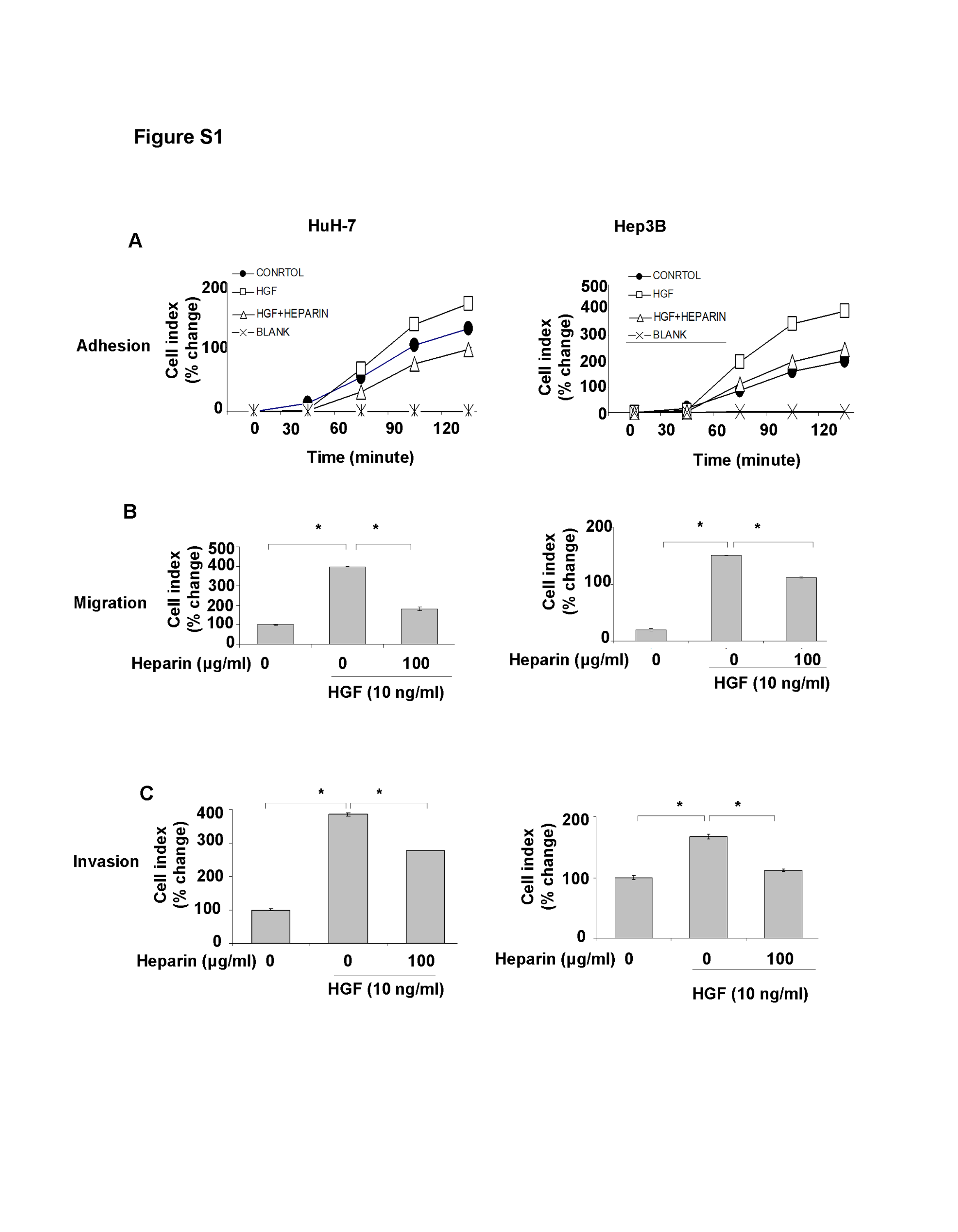

Supplement: Figure S1 — Heparin inhibits HGF-induced adhesion, migration and invasion in HuH-7 and Hep3B HCC cell lines. The effect of heparin on HGF-induced migration and invasion of HuH-7 and Hep3B cell lines were analyzed by using Roche xCELLigence System. Briefly, serum deprived HuH-7 and Hep3B cell lines were left untreated or treated with HGF in the presence or absence of heparin. Adhesion, migration and invasion assays were then performed. E-PLATES, CIM-plates and Matrigel coated CIM-plates were used for real time adhesion, motility and invasion assays, respectively. In both panels, values are expressed as the ratio of adhesive (1A), migrating (1B) or invading (1C) cells in HGF and/or heparin-treated wells. Bars indicate standard error of the mean (SEM), asterisks (*) indicate statistically significant differences between the indicated groups. (TIF) [file pone.0042717.s001.tif]

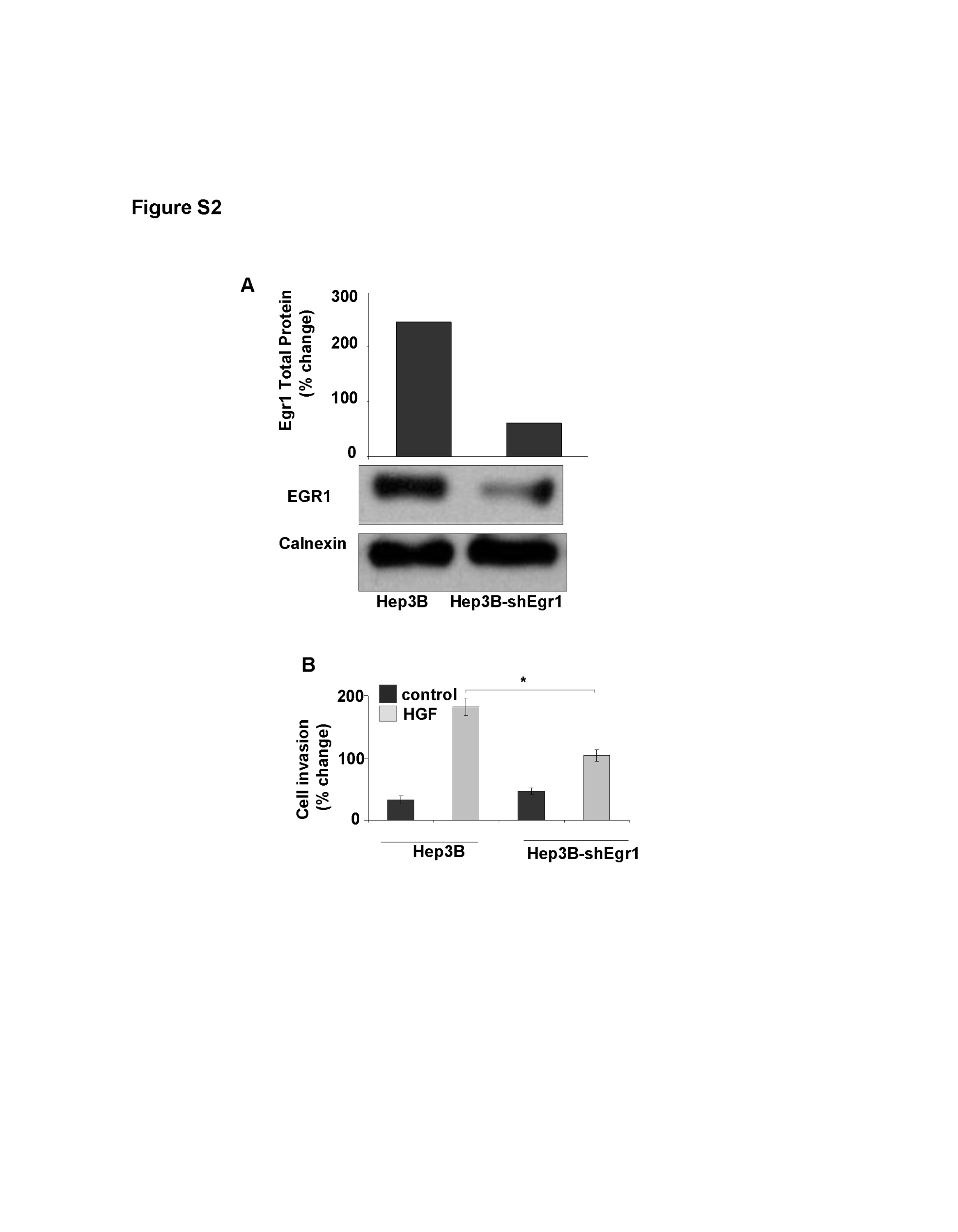

Supplement: Figure S2 — Stable knockdown of Egr1 significantly decreases HGF-induced cell invasion in Hep3B cells. To achieve Egr1 knockdown, Hep3B cells were first transfected with the pSUPER.retro.neo+GFP tet RNAi construct. Stable cells were selected using G418 and marker-selected clones were analyzed for downregulation of Egr1 by western blotting (2A). The graph compares the signal intensities obtained from Egr1 bands. HGF-induced cell invasion in the Egr1 knockdown Hep3B cell line was examined using modified Boyden chamber assays (2B). (TIF) [file pone.0042717.s002.tif]
